# Peripheral blood smear diagnosis of G6PD deficiency in an 83‐year‐old man

**DOI:** 10.1002/jha2.598

**Published:** 2022-12-08

**Authors:** Daniele Sannipoli, Alketa Boletini, Daniela Teresa Clerici, Paola Erminia Ronchi, Fabio Giglio

**Affiliations:** ^1^ Hematology and Bone Marrow Transplantation Unit IRCCS San Raffaele Scientific Institute Milan Italy; ^2^ Immunohematology and Transfusion Medicine Unit IRCCS Ospedale San Raffaele Milano Italy; ^3^ Present address: Onco‐Hematology Unit European Institute of Oncology IRCCS Milan Italy

1

An 83‐year‐old male was admitted to the emergency department for abrupt onset of jaundice and asthenia. Vital signs were in range, and physical examination was unremarkable except for jaundice and hyperchromic urine. Full blood count on admission showed his hemoglobin concentration to be 81 g/L with no alteration in white blood cell (WBC) or platelets count. Biochemical tests were significant for high bilirubin level 417.07 μmol/L (indirect bilirubin 369.53 μmol/L) and abnormal lactate dehydrogenase level 1520 μmol/L. The hypothesis of hemolytic anemia was confirmed by further laboratory tests, which showed undetectable haptoglobin and increased reticulocyte count 196 × 10^9^/L. Direct and indirect Coombs tests were both negative, suggesting a Coombs‐negative hemolytic anemia. A first blood smear did not show schistocytes and the coagulation profile was in order. Medical history was negative for oxidant drugs assumption, prosthetic heart valves, and liver and kidney diseases. Chest radiography was normal, and a complete abdominal echography did not show hepatomegaly or splenomegaly.

Meanwhile, the patient clinical course was complicated by pain and *tightness* in the chest and dizziness and an electrocardiogram was compatible with a non‐ST‐segment elevation myocardial infarction. A new set of lab tests showed severe anemia with 66 g/L of hemoglobin, and the patient undergone blood transfusion. In the suspicion of a hemolytic crisis in the context of glucose‐6‐phosphate dehydrogenase (*G6PD*) deficiency, an accurate anamnesis was collected and the patient confirmed the assumption of fava beans 3 days earlier after a long time. *G6PD* levels could not be assessed overnight, but a second blood smear was significant for bite, blister, and ghost cells (Figure 1 A and B), which are findings compatible with *G6PD* deficiency. Testing for *G6PD* was performed as soon as available and it revealed a significant *G6PD* deficiency (2.7 U/g Hb, normal range 8–14 U/g Hb, overestimated because of low Hb concentration).

**FIGURE 1:**
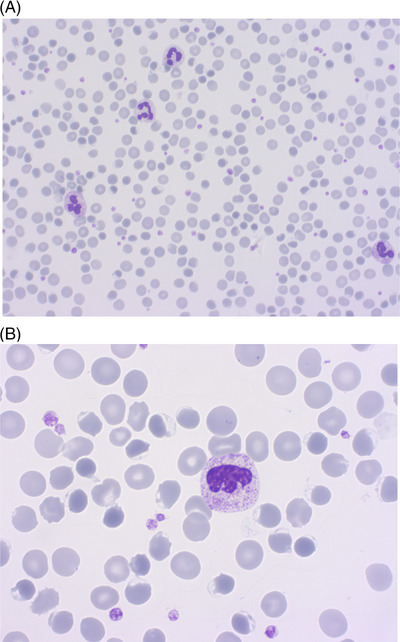
(A and B) Stained blood smears from the patient. Red blood cells anisopoikilocytosis can be appreciated at a lower magnification (A, 40× objective). At a higher resolution (B, 100× objective), blood smear microscopy reveals blisters and ghost cells. Blisters cells present a vacuole on the edge of their membrane, which results from macrophages removal of intracellular detritus. Ghost cells, instead, are a sign of oxidative stress, which in turn determines red blood cells hemolysis after collection.

This is a case of *G6PD* deficiency diagnosed in 83‐yer‐old patient with no previous similar event in an unfavorable setting (emergency department by night). It highlights the necessity of high clinical suspicion of G6PD deficiency in the context of Coombs‐negative hemolytic anemia, even in elderly patients. It is worth noting that different species of fava beans have a different capacity in inducing oxidative stress. Lastly, blood smear can still provide useful morphological data in supporting the diagnosis, regardless the immediate availability of *G6PD* test.

## AUTHOR CONTRIBUTIONS

Fabio Giglio conceived the study; D. Sannipoli, D. T. Clerici, and Fabio Giglio provided all clinical and hematological data; A. Boletini and P. Ronchi performed and analyzed morphology; D. Sannipoli and Fabio Giglio wrote the paper. All authors approved the paper.

## CONFLICT OF INTEREST

The authors declare no conflict of interest.

## Data Availability

Data sharing not applicable to this article as no datasets were generated or analyzed during the current study.

